# Effect of Neuromuscular Electrical Muscle Stimulation on Energy Expenditure in Healthy Adults

**DOI:** 10.3390/s110201932

**Published:** 2011-02-01

**Authors:** Miao-Ju Hsu, Shun-Hwa Wei, Ya-Ju Chang

**Affiliations:** 1 Department of Physical Therapy, College of Health Science, Kaohsiung Medical University, Kaohsiung, Taiwan; E-Mail: mjhsu@kmu.edu.tw; 2 Department of Rehabilitation, Kaohsiung Medical University Hospital, Kaohsiung, Taiwan; 3 Department of Physical Therapy and Assistive Technology, National Yang Ming University, Taipei, Taiwan; E-Mail: shunhwa@ym.edu.tw; 4 Department of Physical Therapy, Graduate Institute of Rehabilitation Science, Chang Gung University, Tao-Yuan, Taiwan

**Keywords:** electrical stimulation, oxygen consumption, calories, energy expenditure

## Abstract

Weight loss/weight control is a major concern in prevention of cardiovascular disease and the realm of health promotion. The primary aim of this study was to investigate the effect of neuromuscular electrical stimulation (NMES) at different intensities on energy expenditure (oxygen and calories) in healthy adults. The secondary aim was to develop a generalized linear regression (GEE) model to predict the increase of energy expenditure facilitated by NMES and identify factors (NMES stimulation intensity level, age, body mass index, weight, body fat percentage, waist/hip ratio, and gender) associated with this NMES-induced increase of energy expenditure. Forty sedentary healthy adults (18 males and 22 females) participated. NMES was given at the following stimulation intensities for 10 minutes each: sensory level (E1), motor threshold (E2), and maximal intensity comfortably tolerated (E3). Cardiopulmonary gas exchange was evaluated during rest, NMES, and recovery stage. The results revealed that NMES at E2 and E3 significantly increased energy expenditure and the energy expenditure at recovery stage was still significantly higher than baseline. The GEE model demonstrated that a linear dose-response relationship existed between the stimulation intensity and the increase of energy expenditure. No subject’s demographic or anthropometric characteristics tested were significantly associated with the increase of energy expenditure. This study suggested NMES may be used to serve as an additional intervention for weight loss programs. Future studies to develop electrical stimulators or stimulation electrodes to maximize the comfort of NMES are recommended.

## 1. Introduction

Weight loss/weight control is a major concern in prevention of cardiovascular disease and the realm of health promotion. Overweight/obesity has emerged as a significant cardiovascular disease risk factor and is also associated with other chronic diseases, such as Type II diabetes and arthritis [1–3]. In addition, excessive weight may predispose to exercise-related injury and discourage people to participate in exercise [4–6], which may further prevent individuals from becoming active and thus, cause a vicious cycle to develop. Therefore, weight management is a key to promote cardiovascular health.

Neuromuscular electrical stimulation (NMES) has been commonly used in physical therapy and rehabilitation to help patients facilitate peripheral circulation, increase muscle power and endurance, and re-educate motor function, *etc.* [7–9]. As health promotion is gaining significant attention, NMES is introduced to augment physical fitness and reduce the risk of heart disease. Clinically, NMES is provided as an alternative to more conventional forms of exercise to encourage increases in physical activity. This is especially true in the case of those who are unable to engage in physical exercise or have barriers to participation, such as individuals with stroke or spinal cord injury (SCI). For example, NMES has been used to help individuals with SCI exercise or passively move their extremities and found to significantly improve their aerobic capacity [10,11]. Other identified health benefits of using NMES in promoting exercise include improved muscle strength/endurance, enhanced peripheral circulation, attenuated bone mineral density loss, improved body composition, more efficient and safer cardiac function, and cardiovascular, and pulmonary training adaptations [12–15].

In addition to aiding in promoting exercise, another common application of NMES is associated with burning fat in that NMES is given on unloaded muscles, *i.e.*, without loading limbs or joints, when an individual is at rest. Commercially, NMES is claimed to be able to facilitate fat burning and has been used to serve as part of weight loss/control programs. It is hypothesized that NMES can enhance energy consuming, considering that NMES induces muscle contraction and increase fat utilization.

Muscle contraction can be viewed as a process converting chemical energy into mechanical work. A body movement is produced by skeletal muscle contraction that substantially increases energy expenditure. The more muscle contracts, the more energy consumes. The energy required during muscle contraction is supplied by three energy systems, ATP-PC, anaerobic glycolysis, and aerobic system. The aerobic energy system uses predominantly fat for energy conversion and thus aerobic metabolism is preferable for weight loss in terms of substrate utilization. Though muscle activity induced by NMES is involuntary and the motor unit recruitment pattern mediated by NMES is different from voluntary muscle contraction, energy expenditure is still essential for muscle contraction. Theoretically, NMES may be used to facilitate energy expenditure and serve as an additional intervention for weight management. However, studies to examine the effect of NMES on this application are very limited [16,17]. Moreover, characteristics of subjects, such as age, gender, and body composition, have been suggested to be associated with energy expenditure during resting or physical activity [18,19]. For example, body weight has been considered as an important factor that influences the energy expended in many forms of exercise like walking or running. Age is negatively correlated with resting metabolic rate, though some part of this effect may be contributed by changes in body fat percentage and fat distribution [18,20]. Nevertheless, whether personal characteristics affect the NMES-induced energy expenditure is still unknown and needs further investigation.

The primary aim of this study was to investigate the effect of NMES at different intensities on energy expenditure (oxygen and calories) in healthy adults. The secondary aim was to develop a model to predict the increase of energy expenditure facilitated by NMES and identify factors (NMES stimulation intensity level, age, body mass index, weight, body fat percentage, waist/hip ratio, and gender) associated with this NMES-induced increase of energy expenditure.

## 2. Methods

### 2.1. Participants

Forty sedentary healthy adults (18 males and 22 females, aged 20–63 years old) with no apparent diseases and no experience of NMES treatment participated. Written consent was obtained from each subject prior to participation in this study. All study procedures received ethical approval from the review committee at the Chang Gung Medical Center. Body fat percentage was calculated based on a 2-compartment model, with Siri Equation [21]:

%Body Fat=(495÷Body Density)−450

Body density (D_b_) was measured by skinfold method with a caliper (Lange, Johnson Diversey Equipment, Cambridge, MD, USA). Skinfold measurements were taken on chest, abdominen, and thigh for males, and triceps, suprailiac, and thigh for females. The sum of three site skinfolds was used to calculate body density with the following equations [21]:

$$\begin{array}{ll} \text{for males:} & \\ & \text{Db}=1.10938-0.0008267(X1)+0.0000016(X1)2-0.0002574(X2) \end{array}$$

where X1 is the sum of chest, abdominen, and thigh skinfolds; X2 is age in years.

$$\begin{array}{ll} \text{for females:} & \\ & \text{Db}=1.099421-0.0009929(\text{X1})+0.0000023(\text{X1})2-0.0001392(\text{X2}) \end{array}$$

where X1 is the sum of triceps, suprailiac, and thigh skinfolds; X2 is age in years.

The waist/hip ratio is the measure of body fat distribution. The circumference for waist and hip was measured by a tape and the waist/hip ratio was calculated. The body mass index was calculated as body weight divided by square of height (in meters). The basic data of the subject is presented in Table 1.

### 2.2. Experimental Procedure

The hand-held muscle stimulator (SA5730, Sanateach Corporation Co., Ltd) powered by three 1.5 V batteries was used in this study. The waveform was biphasic square wave and stimulation frequency was set at 20 Hz. The duty cycle of NMES was on/off = 1:2. The maximum power output of the stimulator was 100 mA. The stimulation intensity included sensory level (E1), motor threshold (E2), and maximal intensity comfortably tolerated (E3).

**Figure 1.**
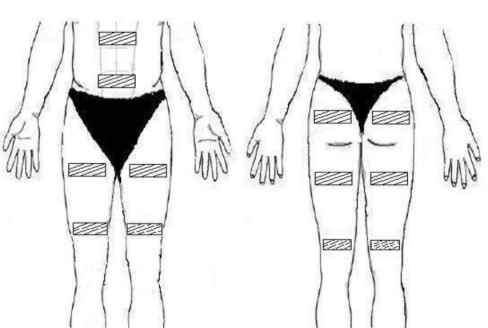
Locations of stimulation electrodes.

The subject was abstained from caffeine at least 24 hours before the testing. The subject sat comfortably and quietly in a chair with back seat. Following skin abrasion with an alcohol-soaked cotton pad, self-adhesive gel electrodes (9 × 12 cm) were placed on abdominal muscles, bilateral gluteal maximum, bilateral quadriceps, and bilateral hamstrings, as illustrated in Figure 1. Prior to testing, stimulation intensities at E1, E2, and E3 were assessed and recorded for each subject. E1 was determined when the subject started to perceive electrical stimulation, while E2 was the stimulation level where muscle contraction could just be visually seen. E3 was the maximal intensity where the subject could still comfortably tolerate. The test session consisted of a 10-minute rest period, followed by 30-minute ES, and then a 10-minute recovery stage. During the 30-minute ES period, each stimulation level (E1, E2, and E3) was provided for 10 minutes. The order of stimulation intensity was randomized. Each subject was free to terminate a test session prematurely if he/she felt uncomfortable for any reason.

During the testing, cardiopulmonary gas exchange was simultaneously evaluated using a facemask and gas analysis system (MetaMax, Cortex Biophysik, Germany) to assess oxygen consumption (VO_2_), carbon dioxide (VCO_2_), and respiratory exchange ratio (RER = VO_2_/VCO_2_). Oxygen consumption (VO_2_) was collected breath by breath. Caloric expenditure was calculated from RER and VO_2_. For each stimulation intensity, the 10-minute data was averaged and used for data analysis.

### 2.3. Data Analysis

The descriptive statistics was employed to analyze mean and standard deviation for each variable. Absolute increases of oxygen consumption and caloric expenditure from baseline were calculated. One-way repeated measures ANOVA was used to examine the differences of NMES intervention at different stages (E1, E2, E3, and recovery) on each variable (oxygen consumption, total calories, and RER). Tukey comparison test was used as the post-hoc analysis. The level of significance was set at 0.05.

Because analyses of factors associated potentially with the absolute increase of calories from baseline elicited by NMES included serial electrical stimulations for the same subject, a generalized estimation equation (GEE) linear regression model using an exchangeable correlation structure was employed. The GEE method was introduced by Liang and Zeger [22,23] to provide standard errors adjusted by multiple observations per person. Independent variables considered in the GEE analysis consisted of NMES intervention stages (E1, E2, E3, and recovery) and the subject’s demographic and anthropometric characteristics, including age, weight, body mass index, body fat percentage, W/H ratio, and gender (male or female). Recovery stage was considered as one of NMES intervention stages due to the interest to analyze the extent of increase in calories at post-NMES. The SAS system software (version 9.1.3, SAS Institute, Cary, NC, USA) was used for statistical analyses.

## 3. Results

**Figure 2.**
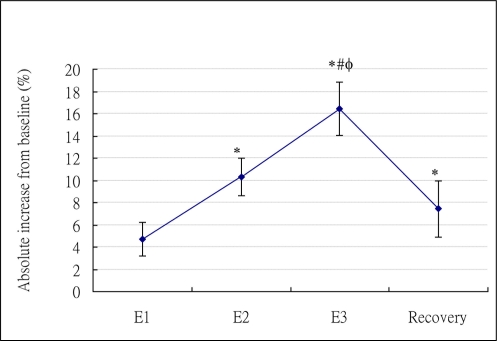
Percentage of absolute increase in caloric expenditure relative to baseline.

The mean and standard deviation of oxygen consumption, total calories, and RER at baseline and different NMES stages (E1, E2, E3, and recovery) were presented in Table 2. Repeated measures ANOVA results revealed that significant main effects of NMES intervention were found on oxygen consumption (P < 0.0001), total calories (P < 0.0001), and RER (P = 0.0002). NMES appeared to increase oxygen consumption and calories at all stimulation levels and also at post-ES stage. However, the post-hoc analysis showed only stimulation intensity equal to or greater than motor threshold significantly elicited the increase on oxygen consumption and calories. Figure 2 presented the percentage of absolute increase of calories relative to baseline for NMES at all stages. RER appeared to be slightly higher during NMES at all stages. However, only RER at E3 was significantly different from baseline (Table 2).

We investigated the departure from linearity of the independent variables related to characteristics of the subject (age, weight, BMI, fat percentage, and W/H ratio) in the GEE model. The quadratic terms were first added in the analysis model. Since none of these quadratic terms were significant, they were excluded from the final GEE model. Table 3 presents the result of the GEE analysis. None of demographic and anthropometric characteristics of subjects significantly contributed to the variations of the absolute increase of energy expenditure from baseline. Only NMES intervention (E1, E2, E3, and recovery) was a significant explanatory variable for the absolute increase in calories from baseline. NMES at E1, E2, E3 and recovery stage induced an increase of 2.96, 6.80, 10.37, and 5.00 units for the absolute increase of calories from baseline, respectively, after adjusting for other variables.

## 4. Discussion

The major finding of this study was that NMES was able to increase energy expenditure and the extent of this increase was aggravated with the increase of NMES intensity. Furthermore, this is the first study to find the energy expenditure was still higher than baseline even after termination of NMES and to identify the relationship between personal characteristics and the NMES-induced energy expenditure.

Much attention has been directed toward the use of NMES for aiding in exercise, while little has been addressed on NMES on physiological responses under resting condition, such as the application of NMES on fat burning. Eijsbouts *et al*. examined whether oxygen consumption could be facilitated in healthy adults (N = 11) during arm-cranking exercise with NMES on legs at maximally tolerated intensity and demonstrated significant increases in oxygen consumption during exercise as well as an approximate increase of 0.08 L/min by NMES at baseline (rest condition) [24]. Banerjee *et al*. investigated NMES at four stimulation outputs (10%, 20%, 30%, and 40% of maximum output) on cardiovascular responses in ten healthy volunteers during rest condition and found NMES significantly increased oxygen consumption, calories, and heart rate. In addition, the physiological responses of NMES were increased with successive increase in stimulation intensity [17]. Those results are supported by our study. However, it is interesting to note that in Banerjee *et al*.’s study, the total calories and oxygen consumption at 40% stimulation intensity were 351 kcal/h and 1.1 L/min, respectively, with the averaged subject’s body weight of 76 kg. Banerjee *et al*. suggested that the NMES-induced level of energy expenditure was similar to the level expected for activities such as walking at 3–3.5 mph. In contrast, in our study, NMES only induced approximately an increase of 76 kcal/h on total calories and 0.26 L/min on oxygen consumption, which was about 30% of that in Banerjee *et al*.’s study after adjusting the factor of the subject’s body weight.

The protocol of NMES stimulation appears to be a major contributory factor to the discrepancy as mentioned above. Banerjee *et al*. attempted to elicit a series of rapid, rhythmical muscle contractions that mimic shivering, with a designed waveform and the stimulation frequency of 4–8 Hz. Instead, using a biphasic square wave and a stimulation frequency of 20 Hz, we intended to induce tetanic muscle contractions without fatigue. The stimulation intensity in their study was quite high, up to approximately 120 mA, while the maximal peak current in our subjects was less than 30 mA, with most of the stimulation intensities ranging from 10 to 15 mA. The stimulation intensity may be the principal determinant for the extent of the increase in energy expenditure. As seen in Banerjee’s study and ours, the energy expenditure induced by NMES is dependent on stimulation intensity. Since our maximal peak current was even less than their simulation level at 10% maximum rated output (30 mA), it would be reasonable to see that the maximal NMES-induced energy expenditure in our study was much lesser. However, only ten subjects were recruited in their study. One may speculate not every individual would tolerate well with their NMES protocol. This speculation is supported in that two out of ten subjects could not tolerate and did not reach the stimulation intensity of 40%. Another two subjects felt minimal discomfort, while the remaining six reported moderate discomfort. On contrary, all of our subjects completed the NMES session without any discomfort. Therefore, though their protocol would facilitate higher energy expenditure, it may only be beneficial to selective individuals who can tolerate high stimulation intensity.

The American College of Sports Medicine (ACSM) suggests an individual to engage in physical activity with accumulation calories expenditure of 250–300 kcal per exercise session (75-kg person) [25]. Based on our results, NMES intervention for one hour could only induce an increase of 76 kcal, if elevated energy expenditure at recovery is not considered. Nevertheless, it may still be practical to implement NMES as part of weight loss programs, especially for those who have very low motivation for exercise or individuals who are unable to participate in exercise or have difficulties to engage in physical activity. First, when an individual is adapted to NMES, a higher stimulation intensity can be tolerated and thus more energy expenditure is induced, indicating shorter NMES intervention duration is needed to cause 250–300 kcal of energy expenditure. Second, the prolonged use of low-frequency NMES treatments may increase muscle mass and improve body composition through improving basal metabolic metabolism, suggesting that daily energy expenditure will be facilitated. Third, evidences have shown that low-frequency NMES may increase muscle capillaries and enhance muscle oxidative ability and thus would possibly improve overall aerobic capacity and exercise performance [26,27]. This might be especially beneficial for individuals with very low motivation for exercise due to low aerobic capacity. Furthermore, implementation of NMES in a weight loss program might have additional benefits, such as the increase of muscle strength and endurance [28].

Previously, no studies followed the effect of NMES on energy expenditure at post-ES stage. Our study is the first to find that the NMES-induced significant increase on physiological responses still last after NMES was terminated. In addition, the extent of this increase at recovery stage was even slightly higher than that at sensory level (E1), though no significant differences were found (Table 2, Figure 2). This phenomenon may be advantageous for the utilization of NMES on weight loss. However, how long does it last can not be answered by this study, since our study only measured 10 minutes after the termination of NMES. Further research is warranted to identify how long this carry-over effect lasts, determine whether a dose-response relationship exists at post-ES stage between NMES intensity and elevated energy expenditure, and understand the underlying mechanism.

Carbondyrate and fat are major fuels used by working muscles. The RER can be used as an index to evaluate substrate utilization (carbonhydrate and fat) for energy production. When fatty acids are principal substrate oxidated, the RER is 0.7, whereas when all carbonhydrate is oxidated, the RER is 1.0 [29]. The smaller the RER, the higher proportion of fat contributes to substrate oxidation. The RER less than 0.85 indicates that at least half of substrate utilization for energy production comes from fatty lipids. In our study, the group mean RER under NMES ranged from 0.81 to 0.83, which was only slightly higher than that of baseline, suggesting that fatty lipids are major fuel and this may be advantageous for weight loss from a substrate utilization standpoint.

Energy expenditure is potentially influenced by characteristics of individuals. To our knowledge, no studies have been done to explore the relationship between personal characteristics and the NMES-induced energy expenditure. In this study, this relationship was investigated using the GEE analysis, which has been used as a suitable strategy to analyze data involving repeated multiple measurements through time [30,31]. According to the GEE model in our study, the absolute increase of energy expenditure from baseline elicited by NMES is not significantly correlated with age, gender, weight, BMI, W/H ratio, or fat percentage, when the stimulation intensity of NMES is controlled (Table 3). Previous studies suggest energy expenditure during walking is greater for obesity people in comparison with normal-weight individuals and higher in obese females in comparison with obese males [19,32]. However, body composition, weight, and gender are not significant contributory factors in our study. These differences may be due to the nature of physical activity. A person must transport his or her body mass during walking, while under non-weight-bearing condition like ours, weight is supported and thus the influence of body weight on energy expenditure is minimized. In addition, one should note that subjects’ demographic and anthropometric characteristics tested in this study can be considered as minor contributory factors, only if stimulation intensity is quantified by perceptions of subjects, *i.e.*, E1, E2, and E3. Furthermore, our subjects tended to be a younger population and most of the subjects were within ideal body fat percentage. Further studies on individuals with a wide spectrum of age or obesity are suggested to confirm that age and obesity do not significantly contribute to variations of increased energy expenditure by NMES.

As shown in Figure 2, it appears that the relationship between levels of stimulation intensity and the NMES-induced increase of energy expenditure was linear. This is also evidenced by the GEE results. According to the GEE model, the NMES-induced caloric expenditure from E1 to E2 (3.84 units) appeared to roughly equal to that from E2 to E3 (3.57 units), indicating a linear relationship existed. We further investigated the linear trend of stages (E1, E2, E3) and statistical significances (P < 0.0001) were found, confirming a linear relationship between stimulation intensity and energy expenditure.

In summary, NMES can significantly facilitate energy expenditure and the energy expenditure post NMES is still higher, which may be advantageous for weight loss. When developing future NMES stimulators for weight management purposes, energy expenditure at post-ES should be estimated and included in the programmed calorie formula to provide accurate information on the NMES-induced energy expenditure. A linear dose-response relationship exists between the NMES-induced energy expenditure and stimulation intensity. Strategies to increase the subject’s tolerance of stimulation intensity are suggested, such as the use of large size stimulation electrodes and adequate skin preparation to minimize electrical impedance. Future studies are recommended to develop electrical stimulators or stimulation electrodes to optimize the comfort of electrical stimulation in order to maximize the benefits and enhance the application of NMES intervention.

## 5. Conclusions and Clinical Applications

This study suggests NMES can increase energy expenditure and the extent of this increase depends on stimulation intensity. In addition, energy expenditure is still elevated even though NMES intervention is terminated. NMES can be used to serve as an additional intervention for weight loss programs.

## Figures and Tables

**Table 1.** Basic data of the subject.

	**Mean (SD)**
**Age (yrs)**	28.2 (11.6)
**Height (cm)**	166.0 (8.0)
**Weight (kg)**	63.0 (10.4)
**Waist/Hip ratio**	0.78 (0.07)
**Body mass index (kg/m^2^)**	22.9 (3.4)
**Body fat percentage (%)**	23.7 (9.8)

**Table 2.** The mean (standard deviation) for oxygen consumption, total calories, and RER during baseline, NMES at different levels of intensities, and recovery.

	**Baseline**	**Electrical Stimulation**			**Recovery**
		**E1**	**E2**	**E3**	
**Oxygen Consumption (L/min)**	0.227 (0.047)	0.238 (0.053)	0.252*# (0.058)	0.265*#ϕ (0.065)	0.242* (0.059)
**Calories (Kcal/hr)**	65.43 (13.64)	68.34 (15.17)	71.89*# (16.26)	76.14*#ϕ (19.13)	68.84* (16.53)
**RER**	0.79 (0.05)	0.81 (0.05)	0.81 (0.06)	0.83* (0.06)	0.79 (0.05)

Note: in Figure 2 and Table 2: E1, E2, and E3 represent NMES at sensory level, motor threshold, and maximal intensity comfortably tolerated, respectively.

* Significances for E1, E2, E3, and recovery *vs.* baseline (Multiple-comparison adjusted P < 0.001);

# Significances for E2, E3, and recovery *vs.* E1 (Multiple-comparison adjusted P < 0.05);

ϕ Significances for E3 and recovery *vs.* E2 (Multiple-comparison adjusted P < 0.05).

**Table 3.** Analysis of GEE.

**Parameter**	**Regression Coefficient**	**Standard Error**	**95% Confidence Limits**		**P**
E1	2.9599	0.9269	1.1431	4.7766	0.0014
E2	6.7960	1.2060	4.4323	9.1596	<0.0001
E3	10.3681	1.7178	7.0012	13.7350	<0.0001
Recovery	5.0039	1.7205	1.6318	8.3761	0.0036
Age (yr)	−0.0459	0.0903	−0.2228	0.1310	0.6108
Weight (kg)	0.0404	0.1765	−0.3054	0.3863	0.8189
BMI	0.8210	0.7881	−0.7236	2.3655	0.2975
Fat percentage (%)	−0.0917	0.1862	−0.4567	0.2733	0.6223
W/H ratio	3.3348	18.1325	−32.2042	38.8738	0.8541
Gender	0.3737	3.7327	−6.9423	7.6896	0.9203
